# Assessing Hydrolytic Activity of Surfactant-Based Nanozymes: Methodological and Kinetic Considerations

**DOI:** 10.3390/nano16020106

**Published:** 2026-01-14

**Authors:** Paolo Tecilla, Paolo Scrimin

**Affiliations:** 1 Department of Chemical and Pharmaceutical Chemistry, University of Trieste, Via Giorgieri 1, 34127 Trieste, Italy; 2 Department of Chemical Sciences, University of Padova, Via Marzolo, 1, 35131 Padova, Italy

**Keywords:** nanozymes, micelles, liposomes, hydrolysis, catalysis, carboxylic esters, phosphoric esters, mechanism

## Abstract

The review critically discusses the methodological approach used to characterize the mechanism and to assess kinetic parameters in catalytic processes promoted by surfactant-based nanozymes. Using the hydrolysis of carboxylic and phosphoric esters as model reactions, it quantitatively analyzes several examples in which the catalytic system consists either of aggregates formed by non-functional surfactants or of surfactants bearing one or more reactive functions, ranging from classical nucleophiles to transition metal ions. This analysis highlights both the importance of the design of the kinetic experiments and of the selection of the appropriate experimental conditions, and the need to apply the correct model and set of kinetic equations in the interpretation of the data, in order to obtain kinetic parameters with true chemical significance. Improper kinetic modeling may lead to misleading rate enhancements and false claims of very high activity of the system studied. The aim of the review is not to provide a general overview of micelle and liposome-promoted catalysis, but rather to offer methodological tools to correctly assess rate accelerations with these systems.

## 1. Introduction

Nobel laureate Jean Marie Lehn has recently highlighted the relevance of self-assembling nanosystems in the scenario of the emergence of life on our planet. He, in fact, states: “[…] (reversible)liquid–liquid phase separation is of particular interest as it introduces interfaces and gives access to compartmentalization and to the emergence of higher-level functions, notably, signal generation, information storage and processing based on constituent redistribution according to the difference in affinities for the macroenvironments. […] Compartmentalization is a hallmark of life emergence and can initiate the appearance of complex behavior […].” [1]. The possibility of creating environments different from that of the bulk solvent, attainable with such aggregates, allows scientists to transform molecules under conditions not achievable in a classical homogeneous solution. This is not much different from what happens in the interior of a protein. Thus, it is not surprising that the study of self-assembling amphiphilic molecules has attracted enormous interest for several decades in an attempt to understand the properties of such aggregates. Focusing at the beginning on the structural aspects of these systems, the research evolved toward the understanding of their interaction with the environment, and, most importantly, for the scope of this review, of their role in catalyzing reactions. Yet, despite this large amount of research, many misunderstandings still exist about these systems. This should not be surprising for their dynamic properties and their multifaceted behavior, as they can interact with hydrophobic molecules and highly polar ions at the same time. The concurrent ability to bind polar and apolar molecules as well ions of these nanosystems makes them excellent candidates for the acceleration of chemical reactions not only based on the trivial argument that higher concentrations correspond to faster rates but also because the local environment can be altered with respect to the bulk solvent and functional groups can be introduced analogously to the side arms of amino acids, the constituents of proteins, that are often involved in the catalytic processes of enzymes [2,3,4,5,6,7]. This review aims to report on established knowledge of the role of these aggregates in catalysis, specifically in hydrolytic reactions, and the best ways to assess rate accelerations with these systems. The relevance of mastering these approaches for the use of these systems in the mimicry of enzymes is obvious, leading researchers to consider these aggregates as rudimentary mimics of their behavior and use the term nanozymes to identify them. This implies that the transformation of the substrate requires first its binding to the catalyst (in this case, a nanocatalyst because of its nanometric dimensions), and the reactions typically occur under pseudo intramolecular conditions. This last point infers that all reactants are eventually confined in a limited space analogous to the enzyme catalytic site. To set the stage for a better understanding of the way of analyzing catalytic processes with these nanosystems, we need to summarize what is well established concerning their physical properties.

## 2. The Dynamic Properties of Amphiphilic Aggregates (Micelles and Vesicles)

**Figure 1:**
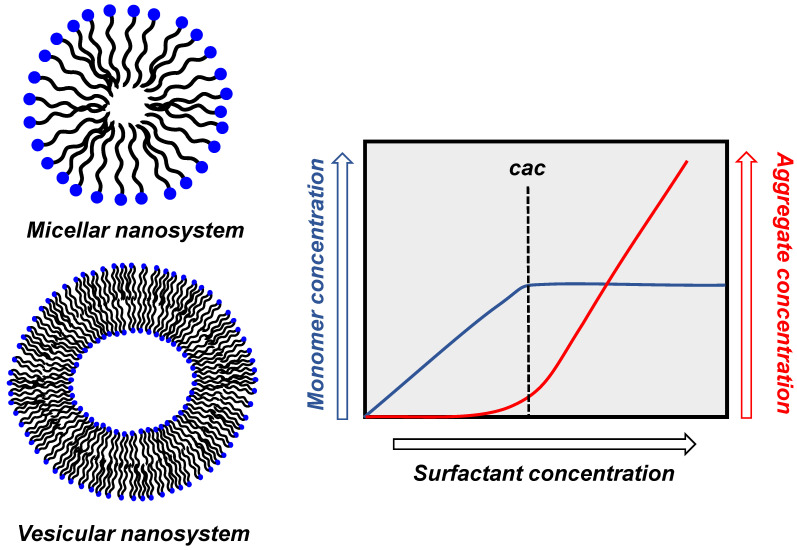
Cartoon representation of micellar and vesicular aggregates and schematic dependence of the concentration of monomer and aggregate as a function of the concentration of surfactant.

The existence of aggregates of amphiphilic molecules depends on the solvent in which they are dissolved. In water, above a critical concentration, the monomers self-assemble to form mostly (but not exclusively) spherical aggregates exposing their polar portion to the bulk solvent. This critical aggregate concentration (cac) is a general term and is specifically called critical micelle concentration (cmc) for micellar aggregates (see below and Figure 1). The term cmc can be replaced by cac without any misconception [8]. Most frequently, two types of spherical aggregates are formed: micelles or vesicles (Figure 1). Micelles are simple systems (a few nanometers in diameter) in which the hydrophobic portion of the amphiphile is located in the interior surrounded by its polar portion. Vesicles are more complex systems. The simplest of them is constituted by a bilayer of amphiphiles, entrapping in its interior an aqueous pool. The nature of the aggregate formed is related to the structure of the hydrophobic moiety [9,10]. For instance, amphiphiles with a single hydrocarbon tail typically form micelles, while those with two of them form vesicles. Often, this bilayer is compared to a biological membrane, of which it represents a minimalistic example. The bilayer “membrane” may insulate the internal water pool from the bulk solvent so that its composition may be quite different from the bulk, analogously to what happens in biological cells. More complex vesicles are composed of several of these bilayers (multilamellar vesicles) [11].

The cac corresponds to the maximum concentration of monomers (i.e., non-aggregated molecules) present in the solution. Above that concentration, aggregation starts with the formation of oligomeric clusters that eventually reach the maximum equilibrium number of them per single aggregate, dictated by their structure and solvent composition. The process is cooperative as aggregate formation, and once the cac is reached, it proceeds exponentially with the increase in the concentration of the monomers. Upon increasing the total concentration of amphiphile, its concentration at the monomeric state does not increase, while the concentration of aggregates increases. It is essential to note that the cac is not an intrinsic property of a given amphiphile, but it depends on the presence of other molecules (polar, hydrophobic, ionic) in the solution. This is relevant for studies undertaken at concentrations close to the cac, as the addition of any additive may alter its value, leading to the disappearance of the aggregates or their formation, respectively, by increasing or decreasing it. Because of the dynamic nature of the aggregates, a large excess of additives could lead to their collapse and disruption. The same may happen with the addition of a cosolvent.

Aggregation may also occur in apolar solvents in the presence of small amounts of water. In this case, a water pool is trapped inside the micelle where the polar headgroups of the amphiphile will be located. The hydrophobic portion is, on the contrary, exposed to the apolar solvent. These systems are referred to as inverse micelles [12,13].

The simplest amphiphilic molecules are made by one or more hydrocarbon chains connected to a polar headgroup (cationic, anionic, zwitterionic, or neutral). To this category also belong natural lipids that are the constituents of biological membranes [14]. Membrane lipids are typically anionic, zwitterionic, or neutral. In principle, any amphiphilic molecule may aggregate in an aqueous solution. Because of this, when working with such molecules, aggregation should always be considered to avoid attributing to the monomer properties that, in fact, belong to the aggregate. The amphiphiles are in equilibrium between their aggregate and monomeric states. The rate of exchange of the single molecules between the two states depends on their solubility in the aqueous medium: the lower the solubility, the lower the exchange rate. For relatively highly soluble amphiphiles (characterized by a high cac), the exchange rate is very fast, almost diffusion-controlled [15]. For poorly soluble ones (very low cac), the exchange rate may become very slow. In some cases, it may take several hours to reach equilibrium [16]. This usually occurs for amphiphilic molecules that aggregate with the formation of vesicles. It is, in fact, very common to prepare these types of aggregates by using sonication to speed up the process. In such circumstances, they are quite often “out of the equilibrium” once the sonication is switched off. For practical purposes, this is not an issue as the shift to the in-the-absence-of-sonication equilibrium is so slow that it is not reached in the time frame of the experiments performed. Selected properties of micellar and vesicular aggregates are summarized in Table 1.

Finally, considering what drives the aggregation process, it must be said that the matter has long been debated. The most accepted current interpretation calls for an important entropic contribution from the release of water molecules upon aggregation and disfavors the prevalence of an enthalpic component [17,18].

## 3. The Interaction of Amphiphilic Aggregates with Neutral or Charged Molecules and with Ions

One of the paradigms of enzyme catalysis is the binding of the substrate to the protein catalytic site, where its transformation will eventually occur. For this reason, when analyzing surfactant aggregates as enzyme-mimetic systems, it is important to understand the interaction that is established between the substrate and any other species possibly involved in the catalytic process.

Any aggregate (micelle or vesicle, for instance) will generate a pseudo-phase [19] in the water solution where it is dissolved, comprising a hydrophobic region, underneath the surface, and a polar one, on the surface where charged (or very polar) species are solvated by aqueous molecules. The aggregation process is obviously associated with an increase in the local concentration of monomers as they are no longer homogeneously dissolved in the aqueous solution but only in the smaller volume of the aggregates. Furthermore, aggregates of amphiphilic molecules are multivalent systems as each of them is composed of several monomers, from ca. 20–100 for micelles to several thousand for vesicles. A hydrophobic molecule present in an aqueous medium will favorably partition into the less polar region of the aggregate [20]. This is similar to what happens when this molecule binds to a unimolecular receptor (host-guest interaction) or a lipophilic substrate binds into the hydrophobic pocket of an enzyme. There are, however, several differences. Two are the most relevant ones. First, an enzyme or a synthetic molecular receptor typically binds a single molecule regardless of the conditions under which the experiment is performed. On the contrary, an aggregate may bind up to several copies of a hydrophobic molecule with a stoichiometry that depends on the conditions of the experiment. Thus, in the presence of a large excess of aggregate, a single molecule per aggregate will be bound, while in the presence of an excess of hydrophobic molecules, several of them will bind to the same aggregate. It is difficult to predict how many of them. However, upon increasing the concentration of added hydrophobic molecules, eventually, the aggregate will start changing its properties and possibly collapse. Second, the mode of binding of a host molecule by a molecular receptor, and even more by an enzyme, is controlled by its molecular structure. This may lead to selective binding. In the absence of specific functional groups present on the amphiphilic monomers of the aggregate, the “recognition” of the host by the aggregate is based almost exclusively on its hydrophobicity, with very little (if any) structural specificity. This is also why an aggregate like a micelle is referred to as a pseudo-phase (see above), like a droplet of organic solvent in an aqueous environment.

Before discussing the details for the determination of the binding of a substrate to an aggregate it is worth recalling a key point in determining an affinity constant: the conditions [host] << [guest] or [guest] << [host] (depending on which species is kept constant during the experiment, host in the first case, guest in the second one) must be always met so that the assumption: [*species varied*]*_total_* ≅ [*species varied*]_free_ is valid throughout the experiment, particularly for the lowest concentrations data, and *K_D_* (dissociation constant) can be obtained from Equation (1) [21]. In this equation, the “fraction bound” refers to the substrate and should be determined by following the change in a physical property of the latter, like absorbance or fluorescence, for instance, upon binding. It refers to the concentration of the species in defect. The curve representing Equation (1) is the classical binding isotherm (a curve that goes to saturation), and *K_D_* is the concentration of the species varied when the fraction bound is 0.5.

(1) $$Fraction\;bound=\frac{{\left[species\;varied\right]}_{total}}{{\left[species\;varied\right]}_{total}+K_{D}}$$

It is important to note that the above equation does not provide a univocal result. The “fraction bound” depends on the conditions under which the experiment is performed, as will be evident in an example we will consider in Section 4.2. Thus, the determination of the affinity constant of a hydrophobic molecule for an aggregate is not straightforward. Using an excess of aggregate (i.e., the varied species is the aggregate), the binding of a single substrate per aggregate will be determined. On the contrary, using an excess of substrate (i.e., the varied species is the substrate), the average binding constant for the maximum number of substrates bound to the aggregate on saturation of the latter will be determined. Obviously, the two numbers are not only quite different, but the second could be rather difficult to obtain for two reasons. First, as said above, the aggregate structure will be substantially altered by the addition of excess substrate, and second, the condition [substrate] >> [aggregate] could be very difficult to maintain, particularly for amphiphiles with high cac. For correctness, one should use the concentration of aggregates, which is [Aggregate] = ([Amphiphile]_total_ − cac)/N_A_, where N_A_ is the average number of monomers per aggregate (aggregation number) [22]. This is relatively straightforward to determine for simple, small micelles, but almost impossible to know for more complex aggregates (such as vesicles, for instance). For this reason, it is common practice to use the amphiphile concentration and subtract the cac instead of the aggregate concentration. The resulting affinity constant is thus affected by a systematic error and is underestimated.

In the case of charged surfactants (cationic, anionic, or zwitterionic), the interface between the charged groups at the surface of the aggregate will also present the counterions and other ions dissolved in the solution, cations for anionic aggregates, and anions for cationic ones [19]. These ions have different affinities for the surface of the aggregate and have different solvation properties. Therefore, they will alter the properties of the aggregate (cac and size, for instance). Very important ions are protons and hydroxyls, for the reason that they will alter the local pH of the external region of the aggregates. Because of this, even when the bulk solution is buffered at a precise pH, the interfacial region of a cationic aggregate will present a higher pH, while that of an anionic aggregate will be more acidic. The difference in pH between the bulk and the interfacial region can be as high as 2 pH units. The consequence is that a lipophilic pH indicator will report the pH at the micellar interface and not that of the bulk solution: adding a cationic surfactant to a buffered solution will indicate a higher pH, while adding an anionic one will indicate a lower pH. This will affect dissociation equilibria, too. For instance, in a pH 6 solution, *p*-nitrophenol (pK_a_ = 7.15) will be mostly non-dissociated. In the same solution in the presence of cationic aggregates to which it will bind, it will be mostly dissociated. Dissociation, in this case, will be favored not only because of the higher local pH but also due to the positive interaction between the cationic headgroups of the amphiphile and the anionic oxygen, which will add up to the hydrophobic interaction.

After having examined the processes that control the interactions of substrates and other species with surfactant aggregates, we are now ready to venture into the analysis of the hydrolytic process catalyzed by surfactant aggregates. We will first briefly examine the accepted mechanisms that drive the hydrolysis of the two most abundant classes of substrates: carboxylic and phosphoric acid derivatives. Afterwards, we will analyze the possible roles of surfactant aggregates in speeding up the hydrolytic process.

## 4. Hydrolytic Activity in Amphiphilic Aggregates

### 4.1. Overview of the Mechanisms of Hydrolysis of Carboxylic and Phosphoric Acid Derivatives

The mechanisms of hydrolysis of carboxylic and phosphoric acid derivatives have been studied in detail, particularly in the second half of the past century. Although differences exist between derivatives within the same class, the basic concepts are rather similar. Because of this and the popularity of esters of carboxylic and phosphoric acids in catalytic studies, we will use these derivatives as model examples.

**Figure 2:**
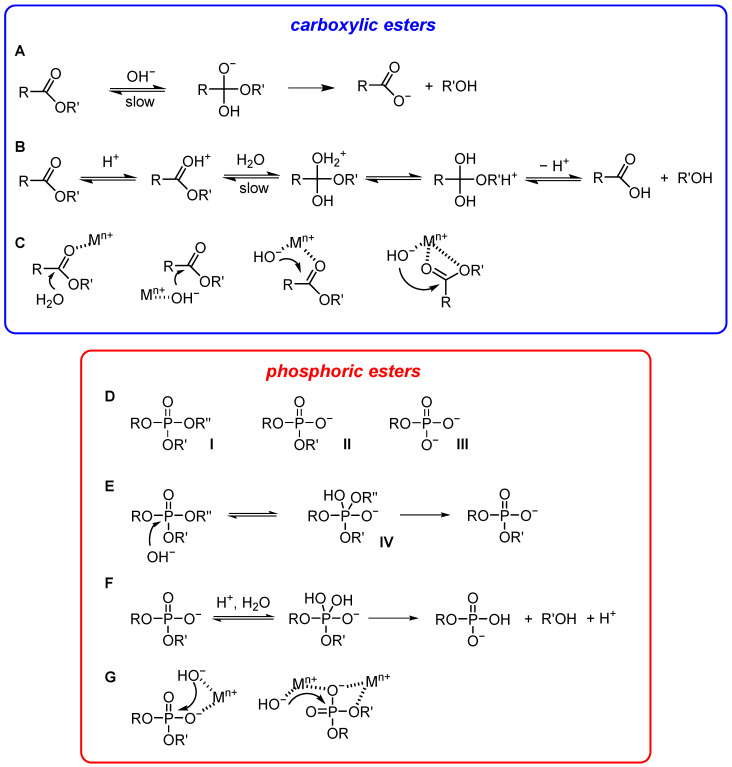
Mechanisms of the hydrolysis of carboxylic (blue box) (**A**–**C**) and phosphoric acid esters (red box) (**D**–**G**).

*Carboxylic esters* [23]. The hydrolysis of these esters is catalyzed by acids or bases. In basic water, the attacking species is the nucleophile OH^−^. In acidic water, the proton catalyzes the reaction by making the carbonyl carbon more positive and therefore more susceptible to attack by the nucleophile (H_2_O). Although Ingold [24] has classified the acid- and base-catalyzed hydrolyses of esters in water into eight mechanisms following these criteria: (1) acid- or base-catalysis; (2) unimolecular or bimolecular, and (3) acyl- or alkyl-cleavage, the most common ones are those reported in Figure 2A,B. Both proceed through a tetrahedral intermediate/transition state (after attack of H_2_O or OH^−^ to the carbonyl carbon) that eventually evolves into the product after the loss of the alcohol. The formation of this tetrahedral intermediate is an equilibrium process, and the ease of its evolution to the hydrolysis product depends on the ability of the alcohol as a leaving group. Poor leaving groups might require acid catalysis for efficient hydrolysis (i.e., assistance in leaving group departure). The role of the proton can be played by a metal ion (typically a transition metal ion) that coordinates to the carbonyl oxygen (Figure 2C). Notice that a water molecule bound to a metal ion is more acidic than H-bonded water. Hence, a metal ion may be a suitable solution for obtaining a higher concentration of OH^−^ at neutral pH for base catalysis (Figure 2C). The metal-coordinated OH^−^ is typically less nucleophilic than free OH^−^. The problem is offset by its much higher concentration (up to more than seven orders of magnitude) under mild pH conditions. Since the metal ion is able to coordinate both a carbonyl and a water oxygen, it may allow for taking advantage of acid and base catalysis contemporaneously (Figure 2C) in the activation of the nucleophile, of the electrophilic carboxyl carbon, and in the stabilization of the leaving group. The replacement of OH^−^ with other nucleophiles does not yield the hydrolysis product but a carboxylic acid derivative. This can be an ester if the nucleophile is an alcoholate (transesterification) or an amide if the nucleophile is an amine, for instance. These intermediates, obtained after the cleavage of the ester substrate, must eventually be cleaved by OH^−^/H_2_O to obtain the hydrolysis product. A relevant nucleophile often found in these two-step catalytic processes is imidazole. Imidazole is a relatively good nucleophile, and because its pKa is ca. 7, it can be easily protonated to become a good leaving group. Most hydrolytic enzymes take advantage of a two-step mechanism for the hydrolysis of carboxylic acid derivatives in which the initial nucleophile is an alcohol (of serine) or an imidazole (of histidine), for instance [25]. The example of serine proteases is discussed at the end of this Section.

*Phosphate esters* [26]. This class of compounds is characterized by three different types of substrates: phosphate triesters (neutral) and phosphate di- and monoesters, both anionic (Figure 2D). Before the introduction of organophosphorus agrochemicals, triesters had no significant presence in the biosphere. Their hydrolysis has become of intense interest because of the need to render harmless stockpiles of some organophosphorus biocides, which include nerve gases as well as herbicides and pesticides, by converting them into unreactive diesters [27,28]. On the other hand, hydrolysis of phosphate diesters is important to manipulate DNA and RNA, key biological polymers in which the monomers are held together by phosphate diester bonds. During a hydrolytic process, a phosphate triester is converted into a phosphate diester, which is hydrolyzed to a phosphate monoester. This latter is eventually converted into inorganic phosphate. Phosphate esters cleavage proceeds through a phosphorane intermediate or transition state, which is the equivalent of the tetrahedral intermediate in carboxylic acid esters cleavage (IV in Figure 2E) [29]. Typically, the spontaneous hydrolysis of a phosphate triester involves water as both nucleophile and general base. The reaction can be catalyzed by better nucleophiles, by stronger general bases, or by both. Hydrolysis of phosphate triesters by hydroxide is a viable process (Figure 2E). Indeed, the destruction of nerve agents by the U.S. government was achieved by incineration and soda treatment. The superiority of the hydroxide nucleophile is much less pronounced with diesters and apparently disappears with dianionic monoesters, which do not react with hydroxide under any conditions. The reactivity of phosphate diesters with hydroxide is up to 8–9 orders of magnitude lower than that of phosphate triesters. Since with any mechanism, the hydrolysis of phosphate esters is characterized by an increase in the negative charge on the phosphoryl group and on the leaving group, catalysis requires the dissipation of such a charge. One way to achieve this is by protonation (Figure 2F). However, extensive protonation requires strongly acidic conditions, where no strong nucleophile can be available. For this reason, phosphate esters are very stable under any conditions. Still, most phosphate esters hydrolyze in strongly acidic conditions, i.e., with weak nucleophiles, faster than at any other pH value. This suggests that neutralization of the ground state and/or of the intermediate/transition state may be the most effective strategy to accelerate the hydrolytic reactivity of phosphate esters. If this is true for phosphate triesters, it is even more important for phosphate diesters. It appears intuitive that a better catalyst than a proton, providing an electrophilic/electrostatic contribution, could be a metal ion. Metal ions play a crucial role in phosphatases and nucleases [30,31]. A metal ion neutralizes the charge of a diester, polarizes the P=O bond, and stabilizes the transition state (phosphate triesters and phosphate diesters). It may also favor the leaving group departure (Figure 2G). Because of these several complementary roles played by metal ions, two (or more) of them are found in catalytic systems, including enzymes.

From the picture emerging from the mechanisms outlined above for the two classes of compounds, it appears evident that, if one wants to accelerate the hydrolysis of these substrates close to the physiological pH there are two possibilities. One is the alteration of the local pH at the reaction locus (as opposed to the bulk solution); the other is the change in mechanism by employing multistep reactions, which utilize other nucleophiles and postpone the hydrolytic step to more labile intermediates. Both approaches are followed by hydrolytic enzymes. By introducing charged species in their catalytic pockets, they alter the local pH and stabilize the charge developing in the transition state. At the same time, other groups are present that may act as nucleophiles, assist in nucleophilic attack, and favor leaving group departure, thus altering the hydrolytic pathways.

For instance, one of the most studied classes of proteases, serine proteases, present in their catalytic site the Ser-His-Asp catalytic triad. Activation of the Ser hydroxyl unit by interaction with the His imidazole forms a strong nucleophile for attack on the carbonyl of the substrate, resulting in a covalently attached acyl-enzyme intermediate and releasing the amine product. Subsequent hydrolysis by H_2_O leads to the acid product and re-establishes the catalytic triad. The anionic transition state is typically stabilized through H-bonding or charge-charge interaction.

In the case of phosphatases, the functional role of metal ions for catalysis has been consistently demonstrated. The experimental data confirm the general mechanistic hypothesis for 2 metal ions-aided phosphoryl transfer in which one metal ion favors the formation of the nucleophile, while the nearby second metal ion facilitates leaving group departure during the hydrolysis. Both metals were suggested to stabilize the enzymatic transition state.

We will see that hydrolytic catalysis with amphiphilic aggregates has been achieved by applying very similar concepts, strengthening the parallel between natural enzymes and amphiphilic aggregates as artificial nanozymes.

### 4.2. Catalysis with Non-Functional Aggregates: Altering the Local pH

We will first discuss the hydrolytic activity of aggregates devoid of functional groups that may be directly involved in the hydrolytic process. With these systems, what affects the activity is the interaction of the aggregate with the substrate and the presence of charged groups. As said above, the charged groups affect the local concentration of ions at the interface aggregate/bulk water, particularly OH^−^ and H^+^, which act as catalysts in hydrolytic reactions. The process requires the binding of the substrate to the aggregate, analogously to what happens with enzymes. For this reason, these systems belong, in a broad sense, to the category of nanozymes. The similarity, however, ends here because, as we have mentioned above, they have no ability to specifically recognize a substrate. Furthermore, they are multivalent and able to bind several copies of the same substrate. This implies that, depending on conditions, several substrate molecules can be processed at the same time, contrary to what happens with enzymes. This is the case when the activity is studied by using an excess substrate following the classical Michaelis-Menten approach. Additional caution in using this approach derives from the effect of such a large amount of substrate on the structure and properties of the aggregate. The opposite approach of using an excess of amphiphile while keeping constant substrate concentration ensures the activity of the aggregate against a single substrate molecule is assessed. A comparison between these two approaches was performed several years ago by Kirby and Hollfelder [32]. To avoid the problem of the alteration of the nature of aggregate with excess substrate, they used an “unimolecular micelle” constituted by an amphiphilic, cationic polymer obtained by alkylating polyethyleneimine with long alkyl groups. The reaction studied was the Kemp elimination of benzisoxazoles. By saturation of the “unimolecular micelle” with the substrate (Michaelis-Menten approach), they obtained (k_cat_)_total_. On the contrary, by keeping constant the concentration of the substrate and increasing the concentration of the “unimolecular micelle”, they also obtained a profile similar to the Michaelis-Menten one, but providing, at saturation, the (k_cat_)_single_, the reactivity of a single molecule of substrate bound to the nanosystem. In their specific case (k_cat_)_total_/(k_cat_)_single_ was 70, implying that the “unimolecular micelle” was able to process at the same time 70 molecules of substrate, assuming the same reactivity in both conditions (which cannot be taken for granted).

**Figure 3:**
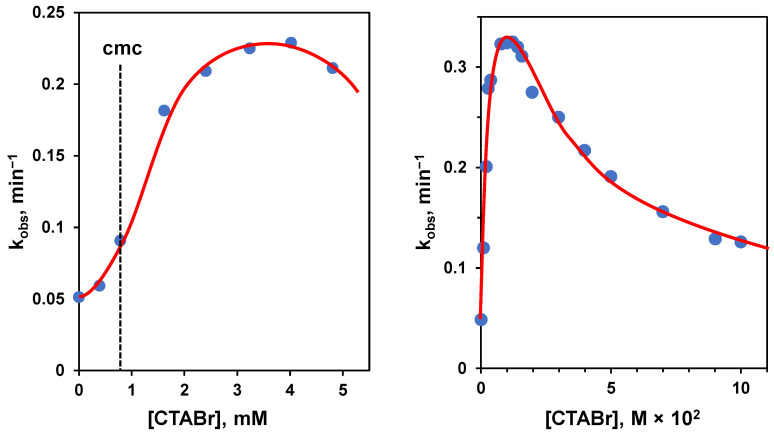
(**Left**): first-order rate constants for the basic hydrolysis of *p*-nitrophenyl hexanoate at 25 °C and pH 10.07 plotted as a function of the concentration of trimethylhexadecylammonium bromide (CTABr). The dotted line indicates the cmc of the aggregate. Adapted from Ref. [33]. (**Right**): first-order rate constants for the basic hydrolysis of *p*-nitrophenyl acetate at 30 °C and pH 9.50 plotted as a function of the concentration of CTABr. Adapted from Ref. [34]. Note that the concentration interval explored in the graph on the right is more than one order of magnitude larger than that on the left.

**Figure 4:**
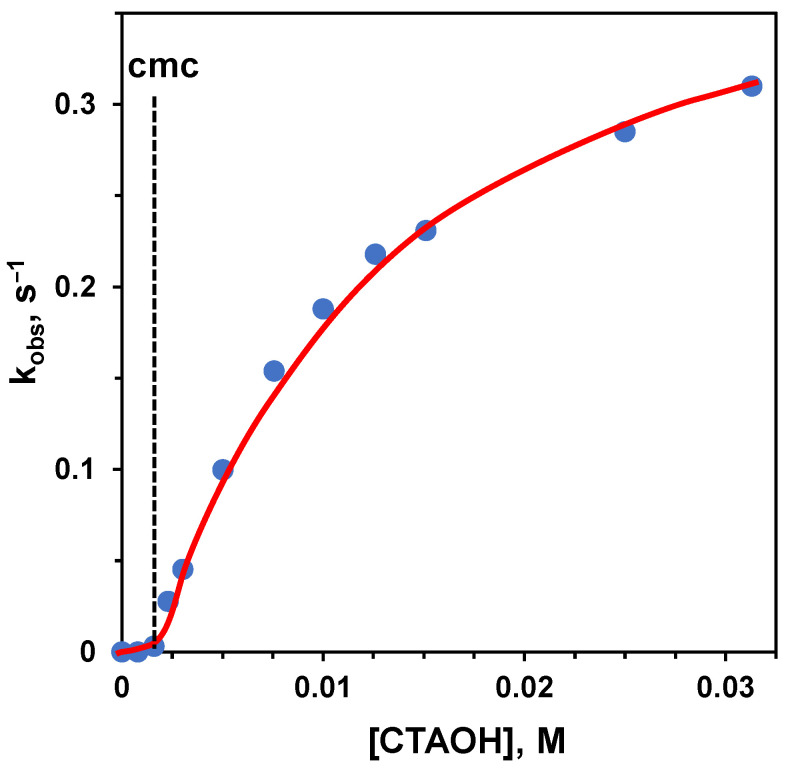
First-order rate constants for the basic hydrolysis of 2-naphthylacetate at 25 °C plotted as a function of the concentration of trimethylhexadecylammonium hydroxide (CTAOH). The dotted line indicates the cmc of the aggregate. Adapted from Ref. [36].

The problems related to using Michaelis-Menten-like conditions and the interest in knowing the reactivity of a single molecule bound to the aggregate could induce one to operate under excess of amphiphile conditions. However, the situation is not simple in this case, too. To understand the problem, we analyze the hydrolysis of a substrate catalyzed by basic conditions: the substrate is *p*-nitrophenyl hexanoate, a lipophilic carboxylate ester, and the aggregate is constituted by micelles of trimethylhexadecylammonium bromide (CTABr), a well-known amphiphile. Since we know that a cationic aggregate will increase the local pH, we should expect that these aggregates will accelerate the hydrolysis rate. Furthermore, the transition state towards the hydrolyzed product is anionic, and its interaction with the cationic headgroups will stabilize it, contributing to the rate acceleration. This is indeed the case. However, by following the reaction upon increasing [CTABr], the authors observed the profile shown in Figure 3 left [33]. Before reaching the cmc, almost no rate increase was observed. After the cmc, the reaction rate increased sharply in association with the formation of the aggregates and their binding to the substrate. Then, instead of plateauing when all substrate was bound, the reaction rate started to decrease. This effect is strictly related to the concentration of the surfactant, and when a large excess of CTABr is added, almost no rate acceleration was observed, as illustrated in the case of the cleavage of p-nitrophenylacetate (Figure 3, right), where the concentration of CTABr was increased up to 0.1 M [34]. The reason for this is that, as the concentration of CTABr increases, so does that of Br^−^, which replaces OH^−^, the catalyst, and inhibits the stabilizing interaction of the anionic transition state with the ammonium ion [19]. Under high [Br^−^], the source of catalysis disappears, and most of the rate acceleration is lost. This implies that the rate constant related to the fully bound substrate can be impossible to achieve experimentally. Note that any added anion will inhibit the reaction. The inhibitory effect will parallel the strength of the interaction of these ions with the cationic headgroups of the amphiphile. Loosely bound anions will be less effective than strongly bound ones. Typically, the inhibitory effect follows the order: NO_3_^−^ > Br^−^ > Cl^−^ > F^−^, which is basically the order of lipophilicity of the anions [35]. Obviously, by using an amphiphile with OH^−^ as the counterion, the inhibitory effect will totally disappear (Figure 4) [36,37]. In this case, the increase in the concentration of the surfactant will correspond to a slight increase in the rate of hydrolysis because of the increase in local [OH^−^].

We can hence summarize the above observations as follows.

A. Using excess substrate (i.e., classical Michaelis-Menten conditions)

(a) Maximum caution must be taken to avoid altering the structure of the aggregate by using excess substrate. To avoid this, typically, relatively large concentrations of amphiphile are used. This implies that the reaction is not studied under optimal conditions, as inhibition by counterion will be present (see above).
(b) The Michaelis-Menten equation will provide (V_max_)_total_ and K_M_ corresponding to the transformation and binding of a number of substrates larger than 1 (possibly up to two orders of magnitude), thus overestimating the intrinsic activity of the system.
(c) The activity value can be (at least in part) corrected by using the [amphiphile] instead of [aggregate] when determining k_cat_. This is an arbitrary and not a rigorous correction.
(d) The equations to be used will be (2) and (3):

v_o_ = (V_max_)_total_ [S]/(K_M_ + [S]) (2)

k_cat_ = (V_max_)_total_/([amphiphile] − cac) (3)

A more realistic value of k_cat_ can be obtained by using the [OH^−^] at the interfacial region (likely 1–2 units larger than that present in the bulk aqueous solution) instead of [amphiphile] − cac, thus the equation will be (4)

k_cat_ = (V_max_)_total_/[OH^−^]_interface_ (4)

B. Using excess amphiphile

(a) The equivalent of the Michaelis-Menten equation should be (5). Note that in this case, since [substrate] is kept constant, the observed rate constant and not the initial rate is determined. This means that the entire kinetic must be followed to obtain the rate constant.
  k_obs_ = k_cat_ ([amphiphile] − cac)/(([amphiphile] − cac) + K_M_) (5)
(b) However, under these conditions, saturation will never be reached (unless a cationic amphiphile is used with OH^−^ as the counterion) as the curve will go through a maximum, and Equation (5) can hardly be used. The implication is that K_M_ (or an affinity constant) should be determined independently.
(c) One may stop at the maximum in collecting rate constants. However, equating k_obs_ at the maximum to k_cat_ will underestimate the value of this latter because of the competing/inhibitory effect of the counterion and the use of the amphiphile concentration instead of that of the aggregate.

As a concise take-home message, we may say that amphiphilic aggregates do alter the local pH leading to significant rate accelerations in hydrolytic processes. However, k_cat_ determined under excess substrate or excess amphiphile conditions cannot be compared: the first will be typically larger than the second, as more molecules are processed at the same time using an excess substrate. Furthermore, when using an excess substrate, the fate of the aggregate must be strictly monitored, while when using excess amphiphile, counterions inhibiting the reaction may lead to falsely higher affinity constants, undermining the correct determination of the kinetic parameters.

For the sake of completeness, it should be said that several theoretical approaches have been reported to correctly fit the reactivity profiles obtained under excess amphiphile conditions [38]. The most successful ones are based on the pseudo-phase model (PP) and its improved variant, the pseudo-phase ion-exchange (PIE). The PP model considers the aqueous medium and aggregate as separate phases with which the substrate is in thermodynamic equilibrium. Since it doesn’t consider interfacial ions, it is mostly valid for spontaneous reactions not involving such ions. The PIE model, starting from the same assumptions as the PP one, also takes into account the competition between nucleophilic (Y^−^) and counterion of surfactant (X^−^), thus managing to fit reactivity data of hydrolysis reactions in a cationic amphiphile (i.e., those going through a maximum) [19,39]. The conclusions drawn based on these analyses are that the observed rate accelerations mostly derive from concentration effects due to the confinement of the substrate and reacting ions in the interfacial volume aggregate/bulk aqueous solution.

### 4.3. Catalysis with Functional Aggregates: Altering the Hydrolytic Pathway

**Figure 5:**
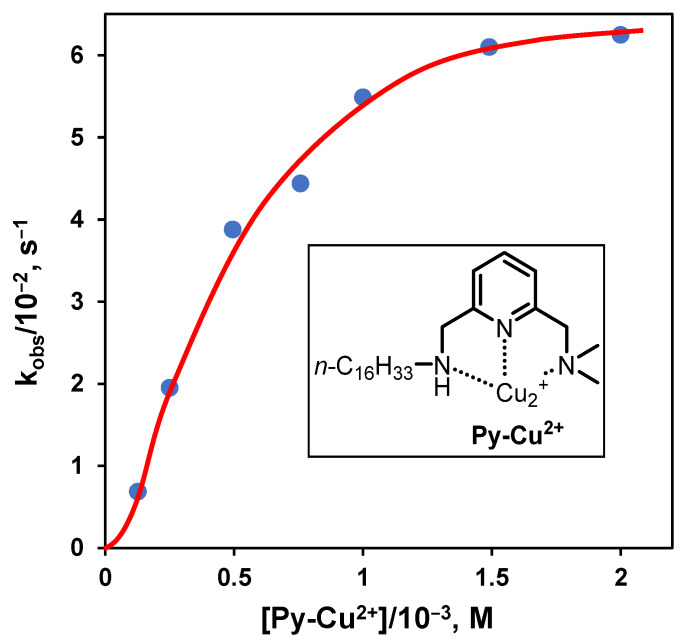
First-order rate constants for the cleavage of PNPDPP plotted as a function of the concentration of the metallosurfactant **Py-Cu^2+^** at pH 9 in 0.01 M CHES. Adapted from Ref. [46].

Although by using cationic amphiphiles, one may obtain significant rate acceleration of base-catalyzed hydrolyses, real enzyme mimicry requires the use of more sophisticated headgroups like those incorporating functional groups present in the catalytic sites of hydrolytic enzymes. This led to a significant amount of research, particularly in the last part of the previous century. Several reviews, book chapters, and even entire books have been published to cover all these studies [40,41,42,43,44]. A recent review comparing hydrolytic surfactant with other supramolecular systems was published by Connal [45]. The key point here, before venturing into more sophisticated systems, is to understand what happens when a functional group (for instance, a nucleophile) is bound to the surfactant. Several nucleophiles have been considered as an imidazole, an oxime, a thiol, and a hydroxyl group bound to the metal center of a metallosurfactant, for example. Operating with such surfactants will not alter scenario A of the previous Section, but affect conditions Bb,c, as the kinetic profile will reach a maximum and never bend downward because of counterion inhibition. One example is illustrated in Figure 5, which reports the kinetic profiles for the cleavage of *p*-nitrophenyldiphenylphosphate (PNPDPP) catalyzed by the metallosurfactant **Py-Cu2^+^** [46]. This implies that Equation (5) can be safely used in this case.

However, the introduction of a nucleophile may lead to its inactivation if it remains acylated after its interaction with the substrate, like a carboxylate ester. If this happens, indicating that the hydrolysis of this intermediate is slower than that of the attack of the nucleophile to the substrate, inhibition is observed in the course of the reaction as the nucleophilic groups of the surfactant get acylated. At this point, the reaction will be controlled by the rate of hydrolysis of the intermediate formed and the turnover of the catalyst. Under these conditions, it is not possible to determine the rate parameters under classical Michaelis-Menten conditions (scenario A of the previous Section). Note that this scenario may also occur in phosphate esters hydrolysis. In this case, a phosphorylated imidazole intermediate will be obtained [47].

**Figure 6:**
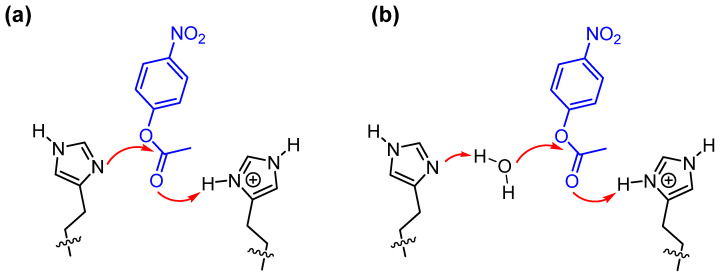
Cooperativity between two imidazoles in the cleavage of *p*-nitrophenylacetate: the non-protonated one acts as nucleophile (**a**) or as general base (**b**) while the protonated one acts as an acid, transferring a proton to neutralize the negative charge developing on the carboxylate oxygen.

Analogously to what happens with natural enzymes, the challenge is to be able to present in the catalytic site both a good nucleophile and an acid. By interacting with the carbonyl (or leaving group, or both) of the substrate, the acid will favor the nucleophilic attack (or leaving group departure) by the nucleophile. Obviously, acid and nucleophile should not interfere with each other to avoid their reciprocal “killing”. This is not trivial to achieve. One of the tricks used by natural systems is that of taking advantage of the same molecule in its protonated (acid) and non-protonated (base) forms. Optimum conditions are obtained at a pH close to the pKa of this molecule, as both forms are equally present. For this purpose, several enzymes have chosen imidazole (present in histidine) as its pKa is around 7, the pH at which these proteins carry out their catalytic process. Indeed, aggregates of imidazole-functionalized molecules have proven to take advantage of this multiple catalysis. This is more likely to occur in micellized polymers [48] or on the surface of ligand-passivated gold nanoparticles [49] than in micelles [50] because of the lower mobility present in these nanosystems. High mobility often prevents cooperativity. The double role of imidazole is shown in Figure 6. More sophisticated systems have been prepared by introducing several functional groups like the catalytic triad present in some hydrolases, consisting of a hydroxyl group, an imidazole group, and a carboxyl group (of a serine residue, of a histidine residue, and of aspartic acid, respectively, in the enzyme) [51]. These groups need to operate cooperatively to achieve maximum catalysis and prevent the build-up of catalytically inefficient acylated intermediates.

It is important to note that catalysis performed with simple nucleophiles is not particularly sensitive to pH once this has been set at a value at which the nucleophile is in its active form. In the case of functional groups performing different tasks and featuring different pKas, there is typically an optimum pH at which the maximum activity of all of them is achieved. This is true for enzymes and functional surfactants as well.

### 4.4. Co-Aggregates of Functional/Non-Functional Systems

The discussion made in Section 4.3 refers to molecules presenting in their structure the lipophilic and hydrophilic moieties (which make them amphiphilic) and the functional group(s) as well. The synthesis of such molecules could be quite demanding. For this reason, scientists have used a mixture of a non-functional amphiphile (like CTABr, see Section 4.2) with a lipophilic molecule containing the proper functional groups [51]. The synthesis of such molecules is usually less complicated. These mixtures are dubbed co-surfactant aggregates. Typically, the non-functional surfactant is present in excess and is not involved in the catalytic process (but for altering the local pH, of course). Such co-aggregates are also interesting because they allow the bypassing of two problems, both under classical Michaelis-Menten conditions. The first one is the possibility to operate under excess substrate also at the lowest concentration of this latter, as the conditions for the formation of the aggregate are guaranteed by the non-functional surfactant, so that the concentration of the catalyst may remain low. The second deals with problem Aa (Section 4.2). Since the excess of substrate will be referred in this case to the lipophilic functional molecule, which is the catalyst and present at a much lower concentration than that of the non-functional surfactant, the risk of altering or disrupting the aggregate will be minimized. For these co-aggregates, all other observations made for functional aggregates (Section 4.3) do apply.

Of course, nothing comes at no cost. Since the binding of the substrate to the co-micelle is usually not specific, the reaction carried out under these conditions will be strongly influenced by the concentration of the functional molecules within the micelle. Unless the morphology of the aggregate is changed by the increase in the lipophilic functional molecules, the reaction rate typically increases linearly with the increase in the concentration of the latter, which is the active catalyst. The interaction between two (or more) functional molecules within the micelle may also lead to deviation from linearity, with the curve bending upward in case of positive cooperativity and downward in case of negative cooperativity. As a matter of fact, the increase in the concentration of the active, functional molecule is the typical experiment to be performed to unravel the occurrence of cooperative phenomena between functional groups and provide hints on the mechanism of the reaction. Classical experiments that are performed to ensure the structural integrity of the aggregates studied are dynamic light scattering or TEM analysis.

## 5. Examples of the Analysis of the Activity of Hydrolytic Reactions Catalyzed by Surfactant Aggregates

### 5.1. Non-Functional Aggregates

**Figure 7:**
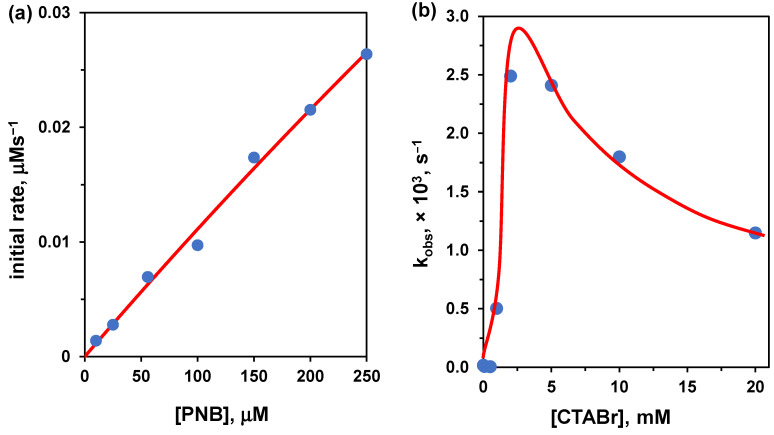
(**a**) Initial rate vs. substrate concentration for the cleavage of *p*-nitrophenylbenzoate (PNB) in the presence of CTABr (0.8 mM) at 25 °C in 0.1 M borate buffer (pH 9.0). The red line is the fitting with the Michaelis-Menten equation (see text). Adapted from Ref. [51] (**b**) First-order rate constants for the cleavage of PNB plotted as a function of the concentration of CTABr at pH 9.18, 5 mM borate buffer, 35 °C. Adapted from Ref. [52].

It is quite difficult to find data on hydrolyses catalyzed by cationic non-functional amphiphiles analyzed by using Michaelis-Menten conditions, as, typically, reactions are performed with excess amphiphile. We found an example for the hydrolysis of *p*-nitrophenylbenzoate reported by Connal et al [51]. The analogous reaction studied in excess of amphiphile was reported by Hojo under similar conditions [52]. Thus, we could compare both approaches, excess of substrate and excess of amphiphile. Our interpolation of the published data [51] with excess substrate (Figure 7) gave the following Michaelis-Menten parameters: V_max_ = 0.352 μM/s, K_M_ = 3066 μM at pH = 9 (100 mM borate buffer), 25 °C, and [CTABr] = 0.8 mM. To determine k_cat_ we have to determine the catalyst concentration. The nucleophile is OH^−^. If we take [OH^−^] = [CTABr]-cac, k_cat_ would be 1.1 × 10^−2^ s^−1^. This is an unlikely scenario as part of the anions present are Br^−^ ions (and borate as well). A more realistic scenario suggests using the local [OH^−^], assuming an interfacial pH = 10 (one unit higher than the nominal bulk solution pH). In this case, k_cat_ becomes 3.5 × 10^−2^ s^−1^. It is worth noting that the CTABr concentration is less than twice the determined cac under the experimental conditions (cac = 0.49 mM), and the Michaelis-Menten fitting is prone to a large error. Hence, these data should be taken with great caution, more as order of magnitude values than correct numbers.

We now analyze the results obtained under excess amphiphile (i.e., keeping constant the substrate concentration and varying that of CTABr). The conditions used [52] were slightly different (pH = 9.18, 5 mM borate buffer, 35 °C) than the above. The curve goes through a maximum and the highest estimated value for k_obs_ is 2.8 × 10^−3^ s^−1^ at [CTABr] = 2.5 mM, which is slightly less than 3-fold the cac under the experimental conditions (cac = 0.91 mM). In taking k_obs_ = k_cat_, we underestimate the real value of k_cat_ because under these conditions, the substrate is not fully bound to the aggregate, as we have anticipated in Section 4.2, Bc.

With the above limitations in mind, we can now compare the k_cat_ obtained under the two different conditions: (k_cat_)_excess substrate_/(k_cat_)_excess amphiphile_ = 12.5. We have used here the more realistic interfacial OH^−^ concentration instead of the amphiphile concentration. This number should not be surprising, as the value obtained under excess substrate represents the contemporaneous hydrolysis of several substrates at the same time, contrary to the single substrate molecule processed under excess amphiphile conditions.

In short, we have shown that the approach used to analyze the kinetic data (excess substrate or excess surfactant) leads to different results. As we have already anticipated (k_cat_)_excess substrate_ > (k_cat_)_excess amphiphile_. This must be kept in mind when comparing results obtained under different conditions.

### 5.2. Functional Aggregates with the Formation of an Intermediate

Many functional groups may act as nucleophiles in hydrolytic reactions. Typically, under these conditions, the acylation of the functional group first occurs, followed by its hydrolysis to complete the overall hydrolytic process. As stated above (see Section 4.3), if the hydrolysis of this intermediate is slower than its acylation, the reaction, following an initial fast consumption of substrate (up to the concentration of the catalyst), enters a slower kinetic regime in which the nucleophile is turned over. This is the case of most imidazole-functionalized cationic surfactants in which the imidazole acts exclusively as the nucleophile. In these cases, reactions performed under excess surfactant or excess substrate provide information about two different processes: the acylation of the nucleophile, under excess surfactant, and hydrolysis of the latter (once the steady-state conditions are reached) by using excess substrate [53]. Because of this, we cannot compare the reactions under excess substrate or excess catalyst, as they refer to two different processes. This is an important caveat for anyone studying functional micelles as catalysts whenever an intermediate is formed.

**Figure 8:**
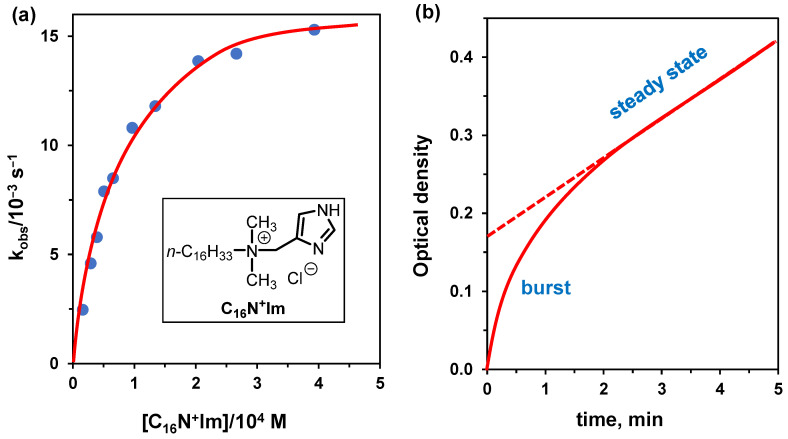
(**a**) Rate-concentration profile for the hydrolysis of PNPH (3 × 10^−6^ M) catalyzed by comicelles of CTABr and C_16_N^+^Im (molar ratio = 6.7) in tris buffer at pH 7.95. (**b**) Kinetic profile for the hydrolysis of PNPH (1.59 × 10^−4^ M) in the presence of C_16_N^+^Im (1.15 × 10^−5^ M) and CTABr (7.80 × 10^−5^ M) in tris buffer at pH 7.95. Adapted from Ref. [53].

We examine, as an example, the cleavage of *p*-nitrophenyl hexanoate (PNPH) by surfactant C_16_N^+^Im (Figure 8) [53]. In this reaction, the imidazole acts exclusively as a nucleophile. The equation to be used is (5) (see Section 4.2) because, contrary to what is observed with non-functional surfactants, the reaction rate goes to saturation as the concentration of functional surfactant is increased, allowing the determination of both K_M_ and k_cat_. Under conditions, tris buffer pH 7.95 and 25 °C, they were K_M_ = 540 μM and k_cat_ = 0.15 s^−1^. The reaction can also be studied using surfactant C_16_N^+^Im in comicelles with CTABr, a condition quite useful to determine kinetic parameters under excess substrate. The results differ very little compared to the homogenous surfactant solution (K_M_ = 470 μM and k_cat_ = 0.14 s^−1^) as the CTABr effect is negligible under the reaction conditions because the imidazole, acting as the nucleophile, is already deprotonated (Figure 8a). As pointed out above, this is the imidazole transacylation reaction and not the hydrolysis of PNPH, as the hydrolysis of the acyl imidazole is slower than its acylation. Under excess substrate (using CTABr comicelles), one is able to calculate the turnover rate (leading to the hydrolysis of PNPH). The k_cat_ for this process is 6.0 × 10^−3^ s^−1^ and is obtained from the slope of the linear portion of the curve (expressed as M × s^−1^) divided by the concentration of the functional surfactant (see Figure 8b). This part of the curve represents the steady-state regime, and this rate is independent of the substrate concentration. On the other hand, the first portion of the curve, named “burst”, reflects the acylation process of the catalyst. The determination of relevant kinetic parameters for enzymes presenting analogous situations was reported many years ago by Bender [54]. The approach is valid for micellar catalysis, too.

We should thus point out that the mechanism of the reaction occurring using a functional surfactant should be known. In particular, if a nucleophile is involved in the formation of the intermediate, it must be assessed whether the latter builds up or if its formation is slower than its hydrolysis. In this latter case, the kinetic analysis in the presence of an excess of substrate or catalyst reports on the same process. If an intermediate doesn’t accumulate, the situation is analogous to the one reported in the subsequent Section.

### 5.3. Functional Aggregates Without the Formation of an Intermediate

Not all functional surfactants present the problem of the build-up of an intermediate. Indeed, scientists have designed sophisticated functional micellar catalysts endowed with complex functional groups resembling the catalytic site of hydrolytic enzymes more closely. Quite often in these cases, the intermediate never accumulates, and the rate constants determined refer to the overall hydrolytic process even when multistep mechanisms are at play.

**Figure 9:**
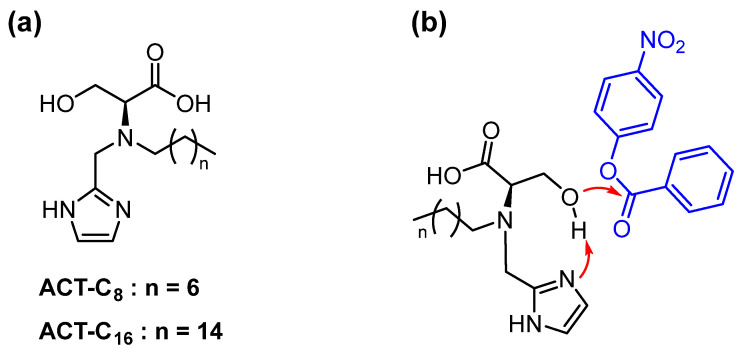
(**a**) Structure of the multifunctional surfactants prepared by Connal’s group. (**b**) Schematic representation of the mechanism of the acylation step with the attack of the hydroxyl group assisted by the transfer of the hydrogen to the imidazole. Adapted from Ref. [51].

A recent example was reported by Connal’s group [51]. Inspired by enzymes that, to undertake catalysis, frequently cluster multiple, complementary chemical units within close proximity in the enzyme active site, they prepared a surfactant catalyst that incorporated the catalytic triad (-OH, -imidazole, and -CO_2_H) we have already discussed (see Section 4.1). They thus obtained trifunctional surfactant molecules (ACT-C_16_ and ACT-C_8_, Figure 9). They were studied as comicelles with CTABr (with 1:333 molar ratio) in the catalysis of the hydrolysis of p-nitrophenylbenzoate. Mechanistic studies supported by QM calculations suggested that the reaction was initiated by the attack of the hydroxyl of the functional surfactant, followed by the fast hydrolysis by OH^−^ of the ester intermediate. Indeed, the experimental evidence did not indicate the build-up of an intermediate as in the reaction involving the imidazole as the nucleophile (see Section 5.2). It was suggested that the imidazole was participating in a relay mechanism, first removing an H^+^ from the alcohol and then delivering it to the leaving group in the final catalytic step. The presence of comicelles that allowed the use of a low concentration of functional surfactant and the absence of a steady-state intermediate legitimized the authors to use the classical Michaelis-Menten approach to obtain the relevant catalytic parameters. Under conditions: 25 °C; 0.1 M borate buffer (pH 9.0), [catalyst] = 2.24 μM; [CTAB] = 0.8 mM, they obtained K_M_ = 716 μM and k_cat_ = 0.28 s^−1^ and K_M_ = 849 μM and k_cat_ = 0.46 s^−1^, for ACT-C_8_ and ACT-C_16_, respectively. In these cases, Equations (2) and (3) were used. In Equation (3), the concentration of the amphiphilic catalyst (ACT-C_16_ or ACT-C8) was used, and the cac was neglected as, under the conditions of the experiments (i.e., large excess of CTABr), it was correctly assumed that all catalysts were bound to the micelles. Using such comicellar system, it was experimentally not feasible to work under conditions of excess surfactant. It should be noted that, because no intermediate accumulates, the reaction performed under the two different conditions would have determined the rate of the same process, in contrast with what happens with functional micelles presenting only an imidazole as the functional group (see above, Section 5.2). We should also mention that old studies with functional micelles featuring an imidazole and a hydroxyl on the same amphiphile (independently performed by Tonellato [55] and Moss [56]) did show the build-up of an intermediate. Although both species were involved, cooperativity was intermolecular rather than intramolecular, suggesting that the relative position of the groups within the same molecule is of fundamental importance for achieving much more efficient intramolecular cooperativity, as in the case discussed here.

If we now compare the examples discussed in Section 5.2 and Section 5.3, we immediately understand the importance of knowing the mechanism of the reaction. The absence of the accumulation of an intermediate (Section 5.3) leads to a much easier interpretation of the data. As a matter of fact, this is real catalysis, while when an intermediate is accumulated, we observe acceleration of the transacylation process but not of the overall hydrolytic one.

### 5.4. Functional Aggregates Where the Substrate Plays a Role in the Aggregation Process

**Figure 10:**
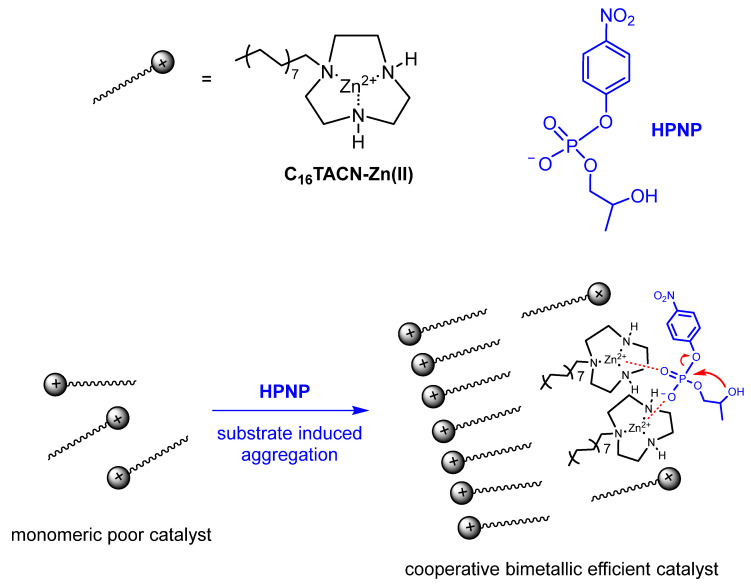
Cooperative catalysis by neighboring C_16_TACN-Zn(II) complexes upon substrate-induced aggregation. Adapted from Ref. [57].

In all the above examples, the conditions ensuring the constant integrity of the aggregate were maintained even in the presence of a large excess of substrate over catalyst because of the use of comicelles. However, the use of comicelles is not always feasible, not least for the “dilution” of the catalyst in the aggregate. Under these “non-ideal” conditions, one should remember that the substrate will alter the aggregation properties of the amphiphile and, possibly, also the structure of the aggregate, as we have discussed in Section 3. A substrate is not much different from a cosurfactant in interacting with the aggregate, and, depending on its concentration, its role will be more or less significant in affecting several aggregate parameters, like the cac, for instance. Nevertheless, catalysis can also be performed under these conditions, provided proper control experiments are performed to determine the dependence of the cac on the substrate concentration and the morphology of the aggregate formed. An interesting example has been recently reported by Chen and Prins [57]. They used amphiphilic Zn(II)-triazacyclononane complexes as catalysts of the cleavage of 2-hydroxypropyl *p*-nitrophenyl phosphate (HPNPP, Figure 10). It is known that this process requires two metal ions for catalysis; hence, monomeric Zn(II) complexes of triazacyclononane are catalytically inactive. Furthermore, earlier work by the Prins’ group had shown that anionic substrates were able to induce the aggregation of amphiphilic Zn(II)-triazacyclononane complexes by substantially decreasing their cac [58]. They argued that lipophilic and contemporaneously anionic HPNPP, would be able to trigger aggregation, thus prompting the cooperativity between two Zn(II) complexes and ensuring the cleavage of the substrate. The substrate would have set up the conditions for its degradation. Obviously, the use of an excess of a cosurfactant to prepare comicellar systems was not feasible here: micelles would have already been formed, and dilution of the catalyst in the aggregate would have played against the cooperativity between two metal complexes. Their hypothesis turned out to be correct. In fact, the cac of C_16_TACN-Zn(II) complex decreased from ca. 93 μM in the absence of HPNPP to ca. 20 μM in the presence of 500 μM HPNPP, with a decrease depending on the concentration of HPNPP present, up to slightly more than 500 μM HPNPP, flattening thereafter. Furthermore, a significant rate acceleration of the cleavage of HPNPP was observed upon aggregation, a clear indication of the ensuing cooperation between two metal ions required for the process. The data also suggested that, apart from decreasing the cac, the aggregate formed switched from micelles to small vesicles upon addition of HPNPP. The morphology of the latter did not change as the [HPNPP] was increased. Michaelis-Menten analysis of the system was feasible under conditions in which the concentration of the substrate was not significantly altering the cac (and, hence, the concentration of aggregates, see below). The values obtained by using Equation (2) were K_M_ = 1.0 mM and V_max_ = 7.2 × 10^−8^ mol s^−1^. By applying Equation (3), a value of k_cat_ = 2.1 × 10^−3^ s^−1^ was obtained, assuming an average cac = 15 μM. Notably, the Michaelis-Menten analysis of the kinetics for C_18_TACN returned a lower value for K_M_ and a similar value of V_max_ compared to C_16_TACN, a reasonable result considering the lower cac of C_18_TACN (under identical kinetic conditions) and, hence, the larger number of aggregates present. This highlights the importance of knowing the dependence of the cac on the concentration of substrate added in the experiment.

This Section evidentiates two of the most peculiar properties of aggregates of amphiphilic molecules: their morphological variability and aggregation flexibility. They should not be overlooked when analyzing the system to avoid making wrong conclusions based on wrong assumptions. The most obvious one is related to the cac: when working at concentrations close to it, it may be that the concentration of the aggregate is very low or that the aggregate hardly exists, with negative consequences in terms of catalysis.

## 6. Conclusions

In this review, we have presented and discussed the correct approach for determining kinetic parameters in the catalysis of hydrolytic reactions mediated by surfactant aggregates, particularly micelles, considering the multiple scenarios one could encounter in the lab. Although this topic emerged in the second half of the last century and has been extensively investigated over the years, the dynamic nature of surfactant aggregates makes kinetic analysis complex and highly sensitive to the experimental conditions employed. Quite often, the Michaelis-Menten analysis of reactivity is compared with data obtained in excess of surfactant without taking into account the fact that intrinsically, the two approaches provide quite different results. Furthermore, the role of counterions in altering the local pH is sometimes ignored, thus providing complex explanations for the observed kinetic data that are totally wrong. Another typical mistake is considering the cac (or cmc) as a constant, ignoring that it varies in the presence of additives (including the substrate). Finally, a poor mechanistic analysis may lead to overlooking intermediates and considering hydrolyses that are in fact transacylations or transphosphorylations.

By analyzing a series of literature examples involving both classical surfactants and surfactants functionalized with reactive groups, we highlighted the necessity of selecting suitable experimental conditions, such as the molar ratio between substrate and surfactant, and applying the correct set of equations to obtain kinetic parameters with true chemical significance. In the case of functionalized surfactants, the situation can be even more complex, as it is essential to consider the behavior of the reactive group during catalysis, where competitive acylation and deacylation processes of the catalyst may occur. Nevertheless, a rigorous analysis and consistent interpretation of the experimental data are still achievable. Although our analysis focused on hydrolytic reactions, the concepts and approaches presented in this review are general and can be applied to other types of reactions. We therefore believe that this review provides a valuable resource for researchers entering the field who wish to investigate micellar catalysis quantitatively and rigorously, while avoiding common methodological pitfalls.

From the point of view of the rate accelerations that can be achieved with the aggregates discussed here, it is clear that a major problem often encountered derives from the limited cooperativity observed. This is a limitation of catalysis using amphiphilic aggregates. Even if the exchange rate aggregate/monomer can be slow, as in vesicles, the interaggregate mobility is typically very fast. For this reason, scientists have developed more efficient systems simply taking advantage of most of the positive contributions of micellar or vesicular aggregates, but devoid of the problem of the fast motion of the monomers that hampers cooperativity. Notable examples are constituted by monolayer passivated nanoparticles [59,60] and polymeric nanoparticles, where it is also possible to select the catalytic site through an imprinting process [61,62]. This is the direction in which the research in this field is evolving, and we expect significant advancements in the near future. In any case, it should be pointed out that although more sophisticated catalysts can be synthesized, these systems combine high efficiency with little synthetic effort and, hence, constitute excellent starting points for obtaining powerful mimics of hydrolytic enzymes [63].

## Figures and Tables

**Table 1:** Selected properties of micellar and vesicular aggregates.

Type of Aggregate	Typical Critical Aggregate Concentration (cac)	Typical Aggregation Number	Typical Size	Typical Monomer/Aggregate Exchange Rate	Effect of the Addition of Lipophilic Molecules	Effect of the Addition of Ions
Micelle	10^−3^–10^−2^ M (ionic amphiphiles); 10^−5^–10^−4^ M (non ionic amphiphiles)	20–100 monomers per micelle	5–30 nm	10^−6^–10^−4^ s	Decreases cac and may change aggregate morphology	Decreases cac up to one order of magnitude (ionic amphiphiles); little effect on non ionic ones
Vesicle	10^−10^–10^−8^ M	10^4^–10^6^ monomers per vesicle	50–500 nm	10^3^–10^5^ s	Decreases stability and may lead to precipitation	Decreases stability and may lead to vesicle fusion

## Data Availability

All the data used for this review are available in the cited documents.
